# Bacterial colonization of bone substitute materials used in oral surgery: mechanisms, clinical implications, and preventive strategies—A narrative review

**DOI:** 10.3389/fmicb.2025.1715632

**Published:** 2025-11-27

**Authors:** Wojciech Popowski, Damian Koseski, Dominika Domanowska, Magdalena Zalewska, Magdalena Popowska

**Affiliations:** 1 Department of Oral Surgery, Medical University of Warsaw, Warsaw, Poland; 2 Department of Bacterial Physiology, Institute of Microbiology, Faculty of Biology, University of Warsaw, Warsaw, Poland

**Keywords:** oral cavity, guided bone regeneration, bone substitute material, oral surgery, colonization, bacterial strains, microbial colonization, bacterial biofilm

## Abstract

The advancement of tissue engineering and the development of novel biomaterials have opened new possibilities for the effective treatment of patients with edentulism and other dental deficiencies, as well as for the prosthetic reconstruction and functional rehabilitation of the stomatognathic system. Bone substitute materials are now widely used in orthopedics, reconstructive surgery, and dentistry to support the regeneration of bone tissue lost due to trauma, inflammation, or tooth extraction. However, surgical procedures within the oral cavity inherently carry a risk of postoperative infection, which can impair healing and compromise treatment outcomes. Unlike natural bone regeneration, bone healing following grafting functions as a repair process that may involve partial resorption of the graft material. Such bone deficiencies can hinder prosthetic reconstruction, making the use of bone substitute materials essential for guided bone regeneration. Bone substitutes can be classified as autogenous, allogenic, xenogenic, or alloplastic, each exhibiting distinct osteoinductive and osteoconductive properties. This review discusses the biological and clinical characteristics of these material groups, with particular attention to their susceptibility to colonization by bacterial strains commonly found in the human oral cavity. It also highlights the risks associated with bacterial biofilm formation and examines its implications for the oral microbiome under dysbiotic conditions.

## 1 Introduction

Bone substitute materials, as well as guided bone regeneration (GBR) techniques, are particularly useful in many surgical specialties where bone tissue regeneration is required during treatment. They are widely applied in orthopedics, reconstructive surgery (e.g., maxillofacial surgery), and in dentistry, especially in oral surgery and periodontology. The range of applications of bone substitute materials in oral and periodontal surgery is constantly expanding. In these cases, bone regeneration is achieved using materials with osteoconductive and osteoinductive properties (Kozakiewicz and Wach, 2020).

Bone substitute materials were initially based on natural or synthetic hydroxyapatite, but today tricalcium phosphate and its composites with hydroxyapatite are commonly used. These materials are typically highly porous to enhance osteoconductivity, support vascularization, and facilitate cellular infiltration. Their primary application is the reconstruction of the alveolar ridge, aiming to restore bone at defect sites. Ideally, grafts should promote the regeneration of vital bone rather than act as inert fillers, gradually resorbing and being replaced by newly formed bone tissue (Uchida et al., 1984; Shimazaki and Mooney, 1985; Verheij et al., 2009). In addition to osteoconductivity, the regenerative potential of these materials depends on their osteoinductive properties, which recruit and differentiate progenitor cells into osteoblasts, accelerating bone formation. Surface characteristics—including micro- and nano-scale topography, chemical composition, and wettability—further influence cell adhesion, proliferation, and integration with host tissue. Thus, optimal bone substitutes combine porosity, osteoconductive and osteoinductive properties, and favorable surface features to achieve predictable and functional bone regeneration (Kozakiewicz and Wach, 2020). Beyond osteogenic potential, material porosity plays a pivotal role in clinical outcomes. High porosity facilitates vascularization, cell migration, and microbial colonization, but may also increase susceptibility to infection in the oral environment. For example, rough and porous xenogeneic surfaces can harbor periopathogens such as *Porphyromonas gingivalis* and *Fusobacterium nucleatum*, while synthetic materials with smoother surfaces may be less prone to bacterial colonization. This microbiological dimension is increasingly relevant, as colonization of graft materials can jeopardize regenerative outcomes (Abushahba et al., 2021; Choi et al., 2023). Postoperative infections associated with bacterial colonization of bone substitute materials constitute a major clinical concern in oral surgery. When pathogens such as *Staphylococcus aureus, P. gingivalis*, or *F. nucleatum* adhere to graft surfaces, they initiate biofilm formation that severely compromises bone regeneration and graft integration. The resulting infection can lead to graft resorption, delayed healing, and even implant failure. Given the constant exposure of oral surgical sites to saliva and microbial biofilms, the development of bone substitute materials with intrinsic antibacterial or bioactive properties has become an essential goal in regenerative dentistry. Antibacterial biomaterials not only inhibit microbial adhesion and growth but also modulate the local immune response and promote osteogenesis. Therefore, comparing the susceptibility of various bone substitute materials to microbial colonization is a key step toward identifying strategies for infection-resistant bone regeneration (Nisyrios et al., 2020; Tu et al., 2023; Yu et al., 2024). Finally, resorption rates vary considerably between materials and strongly influence long-term stability. Autografts and β-tricalcium phosphate (β-TCP) are resorbed relatively quickly, often within months, whereas xenogeneic bovine bone mineral and hydroxyapatite may persist for years. Clinicians must balance this property with treatment goals—rapid resorption may be ideal for defect filling and natural remodeling, while slow resorption helps maintain ridge dimensions for implant placement (Artzi et al., 2004; Hirata et al., 2006; Riachi et al., 2012).

It is critical to recognize that physiological bone healing does not replicate embryonic bone development. Rather, it represents a reparative process with inherent limitations (Mardas et al., 2023). Natural bone remodeling—including resorption—follows tooth extraction and is compounded by mechanical loading from prosthetic reconstructions. These processes result in volumetric loss and morphological alterations of the alveolar ridge in both the maxilla and mandible (Khalifa et al., 2016; Hu et al., 2021). The ensuing bone atrophy disrupts skeletal equilibrium and alters occlusal force distribution, thereby perpetuating resorption. Alveolar ridge deficiency following tooth loss presents a significant therapeutic challenge, often precluding functional and esthetic prosthetic rehabilitation (Hansson and Halldin, 2012; Mardas et al., 2023).

Advances in tissue engineering, and consequently the increasing variety of commercially available biomaterials, have opened new avenues for the effective treatment of patients with tooth deficiencies, enabling subsequent prosthetic rehabilitation and restoration of the stomatognathic system. This review aims to compare groups of bone substitute materials with respect to their susceptibility to colonization by various bacterial strains residing in the human oral cavity, as well as the associated risk of biofilm formation and its implications for the oral microbiome under dysbiotic conditions.

## 2 Bone substitute material in dentistry and periodontology

The application of bone substitute materials today allows for predictable reconstruction of resulting bone deficits. Clinical evidence supporting their use in restoration of alveolar defects, enhancing stability and enabling implant placement was described by (Kim and Kim 2024). The scope of surgical procedures utilizing osteoconductive and osteoinductive bone substitutes is extensive, ranging from post-extraction alveolar ridge augmentation to complex periodontal surgeries, horizontal and vertical alveolar ridge reconstructions, and sinus floor elevation interventions (Elboraey et al., 2025). Rebuilding lost bone tissue is especially critical when implant-prosthetic therapy is planned, as sufficient bone volume is a prerequisite for dental implant placement and subsequent permanent prosthetic restoration (Zhao et al., 2021). The regenerative process of bone comprises multiple contributing factors, of which bone substitute materials are a critical component. Direct influencers of bone healing include initiating and supporting cells—including differentiated, committed, and undifferentiated cells—growth and differentiation factors, and scaffolding materials that serve as carriers for nascent bone (Ansari, 2019).

Bone substitute materials placed into a bone defect—following the creation of the surgical field—initiate the healing process, which comprises four phases: hemostasis, inflammation, proliferation, and maturation. During the hemostatic phase, platelets and clotting factors trigger coagulation and halting bleeding. The inflammatory phase begins approximately 5–6 h post-injury, during which damaged tissues are cleared; inflammatory cells secrete cytokines and growth factors that support the proliferation of fibroblasts and endothelial cells (Cho et al., 2021; Toma et al., 2021; Rodriguez et al., 2024). In the proliferative phase, occurring 2–3 days after injury, contraction of granulation tissue may commence—known as wound contraction—driven by myofibroblasts derived from fibroblasts. Concurrently, increased collagen production supports scarring and leads to contractures during the maturation phase (Fernández-Guarino et al., 2023; Rodriguez et al., 2024).

Bone substitute materials have become indispensable in oral and maxillofacial surgery, periodontology, and implantology, where predictable regeneration of bone tissue is required. These materials differ substantially in their origin, composition, structural properties, and biological behavior, which determines their clinical indications (Table 1).

**Table 1:** Bone substitute materials summary.

**Material type**	**Composition/structure**	**Osteogenic properties**	**Porosity/surface characteristics**	**Resorption rate**	**Susceptibility to microbial colonization**	**Oral applications**	**References**
Autogenous bone	Patient-derived bone; natural mineralized matrix with living osteoblasts and osteocytes	Osteogenic, osteoinductive, osteoconductive	Natural trabecular and cortical architecture	Rapid to moderate	Low if fully covered; contamination possible during harvesting	Ridge augmentation, sinus lifts, critical bone defect repair	Khanijou et al., 2021
Allogeneic bone	Donor human bone, processed to remove cells; preserves mineral matrix	Osteoconductive limited osteoinduction	Trabecular-like structure	Moderate	Moderate; opportunistic pathogens if exposed	Ridge augmentation, sinus lifts, periodontal defects	Santos et al., 2024
Xenogeneic bone (DBBM, Endobon^®^)	Bovine-derived bone, deproteinized, mineral matrix maintained	Osteoconductive	Highly porous scaffold	Slow	High if exposed; early colonizers: *S. mutans, Actinomyces, Veillonella*	Ridge augmentation, periodontal defects	Amid et al., 2021
Alloplasts (HA, β-TCP, BCP)	Synthetic calcium phosphate ceramics; bioactive glasses	Osteoconductive; some osteoinductive	Highly porous; tunable microstructure	Variable (β-TCP faster than HA)	Moderate	Ridge augmentation, sinus lifts, small bone defects	Kim et al., 2025
Collagen membranes (Bio-Gide^®^, CollaTape^®^)	Bovine/porcine-derived collagen, resorbable membrane	Barrier function; supports osteoconduction	Smooth or microtextured surface	Resorbable (weeks to months)	High if exposed	Guided Bone Regeneration (GBR), membrane coverage over grafts	Bunyaratavej and Wang, 2001
Bioactive glasses	Silicate-based glass containing Ca and P	Osteoconductive; may stimulate osteoprogenitor cells	Amorphous or partially crystalline; high surface area	Slow to moderate	Low to moderate	Small bone defects, periodontal regeneration	El-Ghannam, 2005
β-Tricalcium phosphate (β-TCP)	Calcium phosphate ceramic, resorbable scaffold	Osteoconductive	Porous, tunable pore size	Rapid to moderate	Moderate; biofilm formation on surface	Alveolar ridge augmentation, sinus lifts	Roca-Millan et al., 2022
Hydroxyapatite (HA)	Calcium phosphate mineral, similar to bone mineral	Osteoconductive	Crystalline, highly biocompatible, porous	Slow	Moderate; biofilm can form on exposed surfaces	Small bone defects, implant coatings	Arcos and Vallet-Regí, 2020
Autogenous tooth grafts	Processed extracted teeth; mineralized dentin/cementum matrix	Osteoconductive, osteoinductive	Natural dentin tubules; microporous	Moderate	Moderate	Ridge augmentation, alveolar defect reconstruction	Janjua et al., 2022
Plant-derived grafts	Plant-based biomaterials, processed to mimic bone structure	Experimental; mainly osteoconductive	Varies; often microporous	Experimental	Unknown	Experimental; bone defect regeneration	Sharon and Malaiappan, 2025

Autogenous bone grafts (block graft, bone milli, bone scraper, suction device, piezo surgery) remain the gold standard due to their inherent osteogenic, osteoinductive, and osteoconductive properties (Miron, 2024). Harvested from intraoral (mandibular ramus, symphysis) or extraoral donor sites, they contain living osteogenic cells and natural growth factors. However, their clinical use is limited by donor site morbidity, limited availability, and unpredictable resorption rates. Autogenous bone grafts require the creation of an additional surgical site at the donor location, which consequently increases morbidity, the risk of infection, hemorrhage, and potential damage to peripheral nerves (Dantas et al., 2024). Despite these limitations, autografts provide the highest regenerative potential and are frequently used in ridge augmentation and reconstructive maxillofacial procedures (Nkenke and Neukam, 2014; Moraschini et al., 2015). Bone is formed through the processes of osteogenesis, osteoinduction, and osteoconduction. Osteogenesis refers to the direct formation of bone by osteoblasts. Osteoinduction is the ability to stimulate the transformation of mesenchymal cells into osteoblasts, thereby promoting bone growth. Osteoconduction is the process by which new bone is able to adhere and grow along the surface of pre-existing bone (Hoexter, 2002).

Allogeneic grafts (Free Frozen Bone, Freeze-dried Bone Allograft, Demineralized freeze-dried Bone allograft, Deproteinized bone graft), derived from human donors and processed as freeze-dried or demineralized bone matrix, retain osteoconductive and partially osteoinductive capacity but lack viable osteogenic cells. However, allogeneic grafts carry the risk of disease transmission, toxicity related to sterilization processes, variable host immune responses, and limited availability (Lu et al., 2016). They are rich in type I collagen, which constitutes the organic component of bone, providing a matrix that supports cell attachment and proliferation (Rico-Llanos et al., 2021). However, they do not produce inorganic calcium nor provide the mineral scaffold required for full bone regeneration. Unlike autogenous bone, allogeneic grafts do not exhibit osteogenesis; their regenerative potential is mediated solely through osteoinduction and osteoconduction. The porosity and structural integrity of allografts depend on processing techniques, which also influence resorption kinetics. These materials are commonly applied in socket preservation, ridge augmentation, and sinus floor elevation, offering a safe and readily available alternative when autografts are limited or contraindicated (Hoexter, 2002).

Xenogeneic grafts (material derived from corals, animal bone, calcifying algae, or wood), most commonly bovine-derived deproteinized bone mineral, act as osteoconductive scaffolds with a highly porous architecture that closely mimics human bone. Their resorption rate is generally slow, which helps maintain space and volume but may interfere with complete replacement by vital bone. These properties make xenografts particularly useful in sinus floor elevation, ridge preservation, and periodontal regeneration. Studies confirm their long-term dimensional stability, though limited osteoinductive capacity remains a drawback (Hoexter, 2002; Miron, 2024).

Alloplastic materials (calcium phosphates, glass ceramics, polymers, metals), including synthetic hydroxyapatite, β-tricalcium phosphate (β-TCP), biphasic calcium phosphates, and bioactive glasses, represent a broad category of bone substitutes. It is an osteoconductive material composed of very small, unbound calcium crystals, which collectively provide an extremely large surface area (Hoexter, 2002). Hydroxyapatite is highly osteoconductive and structurally stable, but resorbs slowly. In contrast, β-TCP resorbs more rapidly, being replaced by newly formed bone, although excessive resorption may compromise volume maintenance. Biphasic mixtures aim to combine the stability of hydroxyapatite with the remodeling capacity of β-TCP. Their micro- and macroporosity enhance vascular infiltration and cell colonization, a critical factor for osteoconductivity. Bioactive glass, by releasing silica and calcium ions, stimulates osteoblastic activity and angiogenesis, adding an osteostimulative dimension to its osteoconductive nature. These materials are extensively used in ridge augmentation, socket preservation, and periodontal defects (Zhao et al., 2021). Examples of commercially available bone substitute materials in oral surgery are presented in Table 2.

**Table 2:** Examples of commercially available bone substitute materials in oral surgery.

**Type of material**	**Commercial examples**	**Applications/properties**	**Osteoinduction/osteoconduction**
Autogenous bone	Not applicable (harvested from patient: e.g., mandibular ramus, symphysis, iliac crest)	Gold standard Used in ridge augmentation, implant site preparation Maxillofacial reconstruction	Osteoconductive, osteoinductive
Allogeneic bone	Puros^®^ (Zimmer Biomet) MinerOss^®^ (BioHorizons) AlloOss^®^ (ACE Surgical)	Processed human donor bone Used in socket preservation, ridge augmentation, sinus lift	Osteoconductive, partly osteoinductive
Xenogeneic bone	Bio-Oss^®^ (Geistlich) Cerabone^®^ (Botiss) Endobon^®^ (Zimmer Biomet)	Bovine- or porcine-derived deproteinized bone mineral Slow resorption Widely used in sinus floor elevation and ridge preservation	Highly osteoconductive
Synthetic ceramics (alloplasts)	BoneCeramic^®^ (Straumann, HA/β-TCP) Cerasorb^®^ (curasan, β-TCP) Vitoss^®^ (Stryker, β-TCP) OsteoGen^®^ (Impladent, HA)	Variable resorption (β-TCP = faster, HA = slower) Applied in ridge augmentation, periodontal defects, socket grafting	Osteoconductive scaffolds
Bioactive glass	PerioGlas^®^ (NovaBone) NovaBone^®^ Putty	Stimulates osteoblasts and angiogenesis Resorbs into silica and calcium ions Used in periodontal defects and ridge preservation	Osteoconductive, osteoinductive (some of them)
Composite/biphasic materials	MBCP^®^ (Biomatlante, HA/β-TCP) MaxResorb^®^ (Botiss, HA/β-TCP)	Combine stability of HA with remodeling of β-TCP Balance between resorption and volume maintenance Indicated in sinus lift and ridge augmentation	Osteoconductive
Collagen-based matrices with bone grafts	Bio-Gide^®^ (Geistlich, often used with Bio-Oss^®^) Ossix^®^ Plus	Serve as barrier membranes or composite graft carriers Used in guided bone regeneration (GBR) and periodontal regeneration	–
Nano-artificial dental mass (DnP), a mixture of demineralized bone matrix (DBM), calcium sulfate hemihydrate (CSH), curcumin nanoparticles (CU-NP), and silver nanoparticles	DnP	Excellent antibacterial properties with satisfactory mechanical performance	–
Hydroxyapatites	Fisiograft HA Pasta Maxresorb^®^ (bottis) Spher-HA		Osteoconductive

From a regulatory perspective, the use of bone substitute materials in dental surgery and implantology is subject to strict classification within the European Union. In accordance with Regulation (EU) 2017/745 on medical devices (MDR), these materials are considered Class III implantable medical devices, representing the highest risk category. Consequently, their market approval requires a full conformity assessment conducted under the supervision of a Notified Body. The required documentation encompasses detailed technical files, biocompatibility assessments, physicochemical and material testing, functional performance evaluations, and a clinical evaluation report (CER). Only upon successful evaluation by the Notified Body is a CE certificate issued, after which the device is formally registered in the European medical device database (EUDAMED). This regulatory framework emphasizes the critical importance of both safety and efficacy in the clinical application of bone substitute materials.

## 3 Microbial interactions with bone graft materials

### 3.1 Microbiome composition

The oral cavity is a complex ecosystem composed of multiple distinct niches, including the tongue, palate, buccal mucosa, and teeth, each providing a unique environment that shapes resident microbial populations (Morrison et al., 2023). Among the body's microbial communities, the oral microbiome exhibits the second-highest diversity after the gut, encompassing bacteria, archaea, viruses, fungi, and protozoa (Santamaría et al., 2011; Benn et al., 2018). Bacteria dominate these communities and have been most extensively characterized, colonizing both saliva and oral surfaces such as mucosa, teeth, and tongue (Morrison et al., 2023). At the phylum level, a healthy oral bacterial community is largely composed of *Actinobacteria, Fusobacteria, Proteobacteria, Firmicutes*, and *Bacteroidetes*, which together account for nearly all oral bacteria (approximately 96%), with *Fusobacteria* and *Bacteroidetes* being the most abundant (Verma et al., 2018; Nearing et al., 2020; Baker et al., 2024). On the genus level, oral microbial communities exhibit remarkable stability, with 11 genera—including *Streptococcus, Prevotella, Veillonella, Lactobacillus, Actinomyces*, and *Neisseria*—present in over 99% of individuals and representing roughly 78% of total abundance (Morrison et al., 2023). Fungi represent another significant component of the oral ecosystem. More than 100 species have been detected in healthy mouths, with *Candida* spp. being the most prevalent and contributing to the early stages of biofilm formation (Ghannoum et al., 2010; Janus et al., 2017).

However, considerable interindividual variation exists at the species and strain levels, highlighting the personalized nature of the oral microbiome (Nearing et al., 2020). Also, several host- and environment-related variables appear to influence additionally the oral microbiome. Hormonal status is one important factor: cyclical hormonal changes during the menstrual cycle have been associated with shifts in the abundance of the following genera: *Campylobacter, Haemophilus, Oribacterium*, and *Prevotella* (Li X. et al., 2022). Similarly, pregnancy induces distinct microbial alterations, including increased levels of *Neisseria* spp., *Porphyromonas* spp., and *Treponema* spp., whereas non-pregnant women typically exhibit higher abundances of *Streptococcus* spp. and *Veillonella* spp. (Saadaoui et al., 2021). The dysbiosis in oral microbiota is linked to the development of a range of systemic conditions—malignancies such as head and neck, pancreatic, and colorectal cancers, and in chronic inflammatory and immune-mediated disorders including rheumatoid arthritis, systemic lupus erythematosus, hypertension, and Alzheimer's disease (Li Y. et al., 2022; Łasica et al., 2024). Diet represents another key modifier of the oral ecosystem: diets rich in fiber and unsaturated fatty acids (medium-chain, monounsaturated from fish, and polyunsaturated) are associated with greater microbial diversity, whereas frequent consumption of refined carbohydrates and sugars fosters the expansion of certain bacterial groups. The intake of carbonated beverages has been positively correlated with the prevalence of *Bacteroidetes, Gammaproteobacteria, Fusobacterium*, and *Veillonella* (Hansen et al., 2018; Chumponsuk et al., 2021). Environmental factors additionally alter the shape of the oral microbiome—smoking consistently favors the enrichment of anaerobic taxa (Mason et al., 2015), oral hygiene habits substantially influence microbial diversity and community structure (Wade, 2021), or geographic location and climate drives species- and strain-level differences (Mason et al., 2015; Li X. et al., 2022).

In diseases of the oral cavity, including periodontitis and dental caries, a marked shift in the microbial community structure—a state of dysbiosis—has been observed. For instance, the most well-recognized periodontal pathogens comprising the so-called “red complex,” which emerges during the later stages of dental biofilm development, are *P. gingivalis, Treponema denticola*, and *Tannerella forsythia*. Researchers have noted that this “red complex” is consistently identified as part of the climax community within biofilms at sites exhibiting progressive periodontitis. Similarly, in dental caries, other noteworthy bacterial taxa detected in substantial abundance within dental plaque of adults with active lesions include *Streptococcus* spp., *Actinomyces* spp., *Propionibacterium* spp., and *Veillonella* spp. (Holt and Ebersole, 2005; He et al., 2015; Łasica et al., 2024).

Alterations in dentition, including tooth extraction or other surgical interventions within the oral cavity, also exert a considerable impact on the composition and stability of the oral microbiome. Such procedures may disrupt the ecological balance of microbial communities, creating conditions that favor the overgrowth of pathogenic bacteria. In a study investigating the impact of mandibular third molar extraction on the periodontal microbiota and clinical parameters of adjacent teeth, it was found that the microbial community was predominantly composed (90% of sequences) of *Firmicutes, Proteobacteria, Fusobacteriota, Bacteroidota*, and *Actinobacteriota*. Pathogenic bacteria that showed a significant increase compared to the preoperative period included *Prevotella* spp., *Campylobacter* spp., *Porphyromonas* spp., *Pseudomonas* spp., and *Campylobacter gracilis* (Zhang et al., 2024).

In the context of bone grafting and oral surgical procedures, several bacterial taxa are particularly associated with postoperative complications. Opportunistic pathogens such as *S. aureus, P. aeruginosa, E. faecalis, P. gingivalis*, and *F. nucleatum* have been isolated from infected graft sites and peri-implant lesions. These organisms possess virulence factors enabling biofilm formation, resistance to host immunity, and production of tissue-degrading enzymes (Table 3).

**Table 3:** Clinically relevant bacteria associated with bone graft and implant-related infections.

**Bacterial species**	**Clinical relevance**	**Mechanism of virulence**	**Source/typical site**	**References**
*Staphylococcus aureus*	Most common pathogen in graft infections	Biofilm formation, antibiotic resistance (MRSA)	Surgical wound, graft surfaces	Salvi et al., 2008
*Enterococcus faecalis*	Persistent infections, resistant to Ca(OH)_2_	Collagen binding, quorum sensing	Endodontic and graft infections	Flanagan, 2017
*Porphyromonas gingivalis*	Periodontitis, peri-implantitis	Gingipains, immune evasion	Subgingival biofilm	Tzach-Nahman et al., 2017
*Fusobacterium nucleatum*	Bridge organism in biofilm	Coaggregation with early/late colonizers	Oral biofilm, graft surfaces	Chen et al., 2022
*Pseudomonas aeruginosa*	Opportunistic graft infection	EPS production, antimicrobial resistance	Hospital-acquired contamination	Canullo et al., 2015

### 3.2 Bacteria-related implications in oral surgery

Bone substitute materials application clinical success depends not only on osteoconductive and osteoinductive properties but also on their interaction with the complex oral microbiome. The physicochemical characteristics of grafts, including surface roughness and porosity, influence the extent and specificity of microbial adhesion. Colonization of these materials by oral bacteria represents the first step toward biofilm formation, a process that may compromise regenerative outcomes. Understanding the host–material–microbiome triad is essential. Immunological responses to biomaterials, microbial colonization, and biofilm formation represent a dynamic and interconnected continuum that ultimately influences clinical outcomes in implant-based treatments. Optimally designed biomaterials should modulate immune response, resist microbial adhesion, and prevent biofilm maturation to ensure successful bone regeneration and long-term prosthetic function.

Bone substitute materials enabling predictable alveolar bone reconstruction and implant-supported rehabilitation. However, synthetic and allogeneic materials may induce host immune responses as foreign bodies, potentially affecting regeneration outcomes, beginning with protein adsorption, macrophage recruitment, and potential fusion into foreign-body giant cells—events that can compromise integration and healing (Anderson et al., 2008; Moraschini et al., 2020; Albrektsson et al., 2023; ten Brink et al., 2024). Persistent inflammation resulting from this immune response may lead to fibrous encapsulation, thereby reducing the functional stability of the graft or implant (Anderson et al., 2008). The issue of trauma is also not negligible with respect to infections originating at the site of injury. This is associated with the production of pro- and anti-inflammatory mediators, largely by immune effector cells (e.g., neutrophils), which participate in the restoration of homeostasis through the early physiological inflammatory response. Such a response is beneficial in the process of wound healing. However, the occurrence of secondary wound infections following trauma is of particular concern, as these may ultimately progress to sepsis or multiple organ failure (Binkowska et al., 2015). In the context of the oral cavity, the pH of healthy gingival tissues is approximately 6.9, increasing to around 7.2–7.4 under pathological conditions. This shift in pH can modulate gene expression in subgingival bacteria, thereby favoring the proliferation of pathogenic anaerobes such as Porphyromonas gingivalis, which thrives optimally at a pH of approximately 7.5 (Santacroce et al., 2023). The transmucosal design of dental implants introduces a chronic route for microbial invasion, exposing peri-implant tissues to infection and increasing susceptibility to peri-implantitis, which affects up to 25% of implants over time (Reis et al., 2025).

The oral mucosa is colonized by microorganisms, which predisposes surgical sites to infection. This colonization is particularly critical during the healing of newly created operative wounds. Oral wounds cannot be completely immobilized due to the functional demands of the surrounding tissues, and contact with avascular structures limits metabolic exchange during the healing process. Two types of wound healing can be distinguished: primary healing (per primam intentionem), in which tissue regenerates to restore the original structural and functional properties, and secondary healing (per secundam intentionem), where tissue is replaced by fibrous scar tissue, and true regeneration does not occur. Contemporary oral surgery prioritizes primary healing to achieve optimal clinical outcomes (Dragovic et al., 2020). Surgical procedures in the oral cavity carry a significant risk of postoperative infections, which can delay the healing process. Suture materials, commonly used in procedures such as bone regeneration, are recognized as foreign bodies and increase infection risk by serving as surfaces for microbial adhesion. Approximately two-thirds of postoperative infections arise at the incision site, with the presence of sutures further elevating this risk. Microbial colonization of suture materials is a critical factor that negatively influences tissue response to these foreign bodies (Dragovic et al., 2020).

When placed in the oral cavity, bone substitute materials are exposed to the oral microbiome, a highly diverse environment where material characteristics such as surface roughness and porosity largely determine bacterial adhesion. Once microbes adhere, they enter the classic biofilm cycle—adhesion, proliferation, matrix production, and maturation. Bacterial biofilm formation on biomaterials has been widely described by (Li et al. 2023). These biofilms are well-known for their resistance to host defenses and antimicrobial treatments, often leading to graft failure.

### 3.3 Bacterial biofilm formation

Bone substitute materials used in dental implantology are inherently exposed to the oral environment, where their surface properties (composition, roughness, porosity) critically influence microbial interactions. Early bacterial adhesion onto such materials is the first step in biofilm formation, followed by proliferation, matrix production, and eventual maturation—each phase intensifying microbial resilience. A key recent study demonstrated that five representative pathogens (*Escherichia coli, S. aureus, Streptococcus mutans, Enterococcus faecalis*, and *Pseudomonas aeruginosa*) adhere similarly to xenogeneic, allogeneic, and synthetic block bone grafts, indicating that material origin alone does not prevent colonization under *in vitro* conditions (Nisyrios et al., 2020). In this same study, scanning electron microscopy revealed bacterial clusters not only on planar surfaces but also deep within topographic niches, which are features of porous bone substitutes that shelter bacteria from shear forces and host defenses (Nisyrios et al., 2020). These porous scaffolds facilitate vascularization and host cell infiltration, but paradoxically also foster complex microenvironments ideal for bacterial retention. As biofilm develops, bacteria produce extracellular polymeric substances (EPS) that trap nutrients and enable communication, resulting in enhanced protection against antimicrobials. The detailed description on bacterial biofilm formation on implantable devices was extensively reviewed by (Khatoon et al. 2018). The summary of biofilm-colonizing bacteria is presented in Table 4.

**Table 4:** Summary of bone substitution materials and colonizers.

**Material/class**	**Reported colonizing taxa (early/moderate/late)**	**Conditions**	**References**
Block bone grafts xenogeneic Tutobone^®^ alloplastic Endobon^®^ allogeneic human spongiosa	Early adhesion: *E. coli S. aureus S. mutans E. faecalis P. aeruginosa*	*In vitro* study	Nisyrios et al., 2020
Morselized allograft bone/bone allografts	Early adhesion: *S. aureus*	*In vitro* study	Ketonis et al., 2010
HA-based scaffolds/mesoporous HA composites	Tested pathogens: *P. gingivalis F. nucleatum* (late colonizers)	*In vitro* study	Liao et al., 2020
Bioactive glass (S53P4, 45S5)	Tested against: *Veillonella parvula S. aureus* (MRSA) *Streptococcus gordonii*	*In vitro* study Bioactive glasses show antibacterial activity	Zhou et al., 2022
Collagen membranes (resorbable GBR membranes)	Early colonizers: *Streptococcus* spp., *Actinomyces* Periodontopathogens on exposed membranes: *P. gingivalis F. nucleatum*	Membrane exposure leads to rapid microbial colonization and compromised GBR outcomes; both early and late colonizers have been recovered from exposed membranes	Álvarez et al., 2022
Titanium implant surfaces (coated/modified)	Early colonizers: *Streptococcus Actinomyces* Moderate/late: *Fusobacterium* spp., *Porphyromonas* spp. (peri-implant biofilms)	Surface roughness and chemistry influence initial adhesion; antibacterial coatings reduce early colonizer attachment and subsequent biofilm maturation.	Kulkarni Aranya et al., 2017

Furthermore, systematic reviews indicate that bone substitute materials—even when sterilized—can harbor organic and inorganic impurities, such as donor cellular remnants or heavy metal residues, which may further modulate bacterial colonization and immune response (Struzik et al., 2024). These contaminants can provide additional surfaces or niches that favor microbial persistence. Clinical studies of peri-implant disease show that disrupted host–microbe balance near implant surfaces leads to increased presence of periopathogens such as *P. gingivalis, T. forsythia*, and *T. denticola* in biofilms, especially when the implant is exposed to saliva or micro-leakage at the interface (Dhir, 2013; Zhao et al., 2021). These bacteria are known to produce virulence factors that provoke inflammatory responses and stimulate osteoclastogenesis, accelerating alveolar bone loss. The colonization of bone substitute materials and dental implants by oral microorganisms follows a well-defined ecological succession that mirrors classical biofilm development on natural tooth surfaces. Immediately upon implantation or surgical exposure, the biomaterial surface is coated with a conditioning film of salivary proteins and host molecules, which provides binding sites for bacterial adhesion. This initial pellicle is rapidly colonized by early colonizers, predominantly Gram-positive facultative aerobes such as *Streptococcus oralis* and *Actinomyces naeslundii* (Siddiqui et al., 2022). These organisms attach within hours, exploiting surface roughness and porosity of grafts and implants, and they establish the first microbial clusters that serve as a foundation for subsequent biofilm maturation.

As the biofilm matures over the next several days, moderate colonizers integrate into the developing community. Genera such as *Veillonella* and *Fusobacterium* are characteristic at this stage, taking advantage of metabolic by-products of the early colonizers and establishing metabolic cooperation (Dieckow et al., 2024). Importantly, *F. nucleatum* acts as a bridging species, facilitating co-aggregation between early and late colonizers. These interactions gradually lower the redox potential of the biofilm environment, generating anaerobic niches particularly within the porous scaffold of xenogeneic or alloplastic bone substitutes. The final stage of succession involves the establishment of late colonizers, typically obligate anaerobes with pathogenic potential. Species such as *P. gingivalis, Prevotella intermedia*, and *T. denticola* appear after 1–2 weeks of biofilm development, when anaerobic microenvironments are sufficiently stabilized (Dutra et al., 2025). These organisms are strongly implicated in peri-implantitis and graft-related infections, as their virulence factors drive persistent inflammation, osteoclast activation, and progressive alveolar bone loss. Their colonization is therefore not only a marker of biofilm maturity but also a critical determinant of adverse clinical outcomes.

The physicochemical properties of the material play an important modulatory role throughout this process. High surface roughness and interconnected porosity, which are desirable for osteoconduction and vascular ingrowth, also provide protected niches that promote early bacterial adhesion and persistence (Choi et al., 2023). Scanning electron microscopy studies have demonstrated that both smooth and porous regions of bone graft materials harbor dense bacterial clusters, with porosity favoring deep biofilm penetration (Nisyrios et al., 2020). This duality presents a challenge: while porosity supports bone regeneration, it also predisposes to microbial colonization and biofilm maturation.

Surface roughness modifies initial bacterial adhesion: rough titanium or graft surfaces show higher colony forming units in early adhesion assays compared to smoother materials. Surface energy, wettability, and microtopography (micropores, crevices) correlate strongly with bacterial load *in vitro* (Dhir, 2013; Kligman et al., 2021; Zhao et al., 2021). Implant surfaces with micropits, grooves, or porous matrices therefore represent higher risk zones for biofilm initiation if not properly managed. As the biofilm matures, it develops gradients of oxygen tension and nutrient availability; facultative anaerobes near surface transition to anaerobic bacteria deeper inside, mirroring peri-implantitis biofilm structure observed clinically (Dhir, 2013; Zhao et al., 2021). These anaerobes produce enzymes and toxins that degrade extracellular matrix and bone tissue, while also evading immune detection.

The intimate placement of bone grafts in direct contact with host tissue means that biofilm can interfere with graft integration, decreasing bone-to-material contact and promoting micro-motion, which further impedes healing. The presence of bacterial biofilms on grafts often is linked to delayed healing, graft failure, or need for graft removal documented in both animal and human models (Nisyrios et al., 2020; Zhao et al., 2021). Understanding the sequential process—from adhesion to biofilm maturation—on graft materials is thus essential for advancing long-term outcomes in implant-prosthetic dentistry.

## 4 Biofilm formation preventive approaches

The human oral cavity is densely colonized by microorganisms, creating a significant risk of contamination during dental and maxillofacial surgical procedures. Early postoperative infection control is critical, as disruption of initial colonizers can prevent or delay the establishment of pathogenic late colonizers. Contamination of grafts with oral bacteria, particularly *S. aureus*, which is frequently implicated in bone infections, may lead to surgical site infection and impaired healing.

Optimal protection against infection is generally achieved through appropriate antibiotic prophylaxis. Preoperative administration of 2 g amoxicillin 1 h prior to implant placement or procedures involving bone substitute materials has been recommended and is considered effective in reducing postoperative infection risk (Milic et al., 2021; Remschmidt et al., 2023). However, oral antibiotics can sometimes be ineffective, and local applications may present an unfavorable balance between dosing efficacy and potential toxicity, with the additional concern of promoting antibiotic resistance (Kolmas et al., 2014; Abe et al., 2022; Senthil and Çakir, 2024; Łasica et al., 2024). Evidence on the benefits of adjunctive antibiotic therapy in bone regeneration is mixed; some studies report no significant advantage over prophylactic “one-shot” administration (Lyons et al., 2008).

Surgical protocols that minimize graft exposure and ensure optimal soft tissue closure further limit microbial colonization. Preoperative oral decontamination using antiseptic mouth rinses—chlorhexidine (0.12–1%), cetrimide (1%), or povidone-iodine (1%)—significantly reduces bacterial load and decreases postoperative infection risk (Kosutic et al., 2009; Johnson et al., 2015). During sinus floor elevation procedures, autogenous bone particles collected during access preparation (e.g., using bone filters) are susceptible to contamination. Strict aspiration protocols, irrigation with saline or antiseptic solutions, and consideration of prophylactic antibiotics reduce bacterial exposure, including pathogens such as *Aggregatibacter actinomycetemcomitans* and *P. gingivalis* (Manor et al., 2015; Purcz et al., 2015).

Sinus lift procedures are a well-established technique, performed for over 30 years with a low complication rate. In a retrospective analysis of 202 procedures in 127 patients, the most common intraoperative complication was Schneiderian membrane perforation (25.7%), which did not correlate with postoperative complications. Observed postoperative complications included wound infection, abscess formation, or dehiscence requiring drainage (9 cases) (Moreno Vazquez et al., 2014). Potential complications may also involve acute or chronic maxillary sinusitis, which can be resistant to pharmacological treatment (Chirilǎ et al., 2016; Jiam et al., 2017). Similarly, infection at graft sites in socket preservation procedures may lead to failure to maintain the alveolus, requiring additional ridge augmentation before implant placement (Mazzucchi et al., 2020).

In reconstructive surgery, perioperative infections around biomaterials remain a major challenge (Kolmas et al., 2014; Senthil and Çakir, 2024). Infections in the alveolar processes of the maxilla or mandible can result in graft or material loss, procedural failure, and preclusion of subsequent implant-prosthetic rehabilitation. Antimicrobial strategies include the use of autologous platelet-rich fibrin (A-PRF), which provides growth factors for bone regeneration and immune cells with antimicrobial and anti-inflammatory properties (Caruana et al., 2019; Popowski et al., 2025). Additionally, surface modifications of implants and bone substitute materials—such as smoother finishes, bioactive coatings, controlled porosity, and antimicrobial release—can limit bacterial adhesion and biofilm formation, promoting successful regeneration over infection-driven failure.

Various bone substitute materials have been studied for their antimicrobial properties and regenerative potential. Freeze-dried bone allograft (FDBA) is successfully used in the treatment of juvenile periodontitis defects, often combined with local and systemic tetracycline therapy (Mabry et al., 1985). In sinus lift procedures, the addition of metronidazole to FDBA improved graft homogeneity and bone density, whereas control groups without metronidazole showed heterogeneous bone formation and air bubbles in graft structures on early CT scans, possibly indicating anaerobic contamination (Choukroun et al., 2008). Similarly, incorporation of doxycycline (4%) or clindamycin into demineralized freeze-dried bone allograft (DFDBA) has been shown to reduce bacterial counts (e.g., *P. gingivalis*) in periodontal pockets, though statistical significance compared to controls is variable (Lyons et al., 2008; Deepak et al., 2020). Autolyzed, antigen-free allogenic bone (AAA) demonstrated improved intrabony defect regeneration compared to controls, with low detection of *P. gingivalis* and no *A. actinomycetemcomitans* after 3 years (Flemmig et al., 1998). A summary of available data on the antibacterial properties of bone substitute materials is presented in Table 5.

**Table 5:** Antibacterial properties of bone substitute materials.

**Material name**	**Pubmed record**	**Antibacterial activity reported**	***In vitro/in vivo***	**Antibacterial activity assement**	**Bone substitute addition**	**References**
**Allogeneic bone**						
FDBA (freeze- dried bone allograft)	Yes	Studies evaluating the effect of antibiotics	*In vivo*	Not tested	2 ml of 0.5% metronidazole solution	Choukroun et al., 2008
DFDBA (decalcified freeze-dried bone allograft)	Yes	Studies assessing antibiotics use for prevention of colonization	*In vivo*	*P. gingivalis*	Clindamycin	Deepak et al., 2020
AAA (autolyzed antigen extracted allogenic bone)	Yes	Periodontal defect treatment	*In vivo*	*P. gingivalis*	None	Flemmig et al., 1998
DBM (demineralized bone matrix)	Yes	Studies evaluating the effect of antibiotics	*In vitro*	*A. naeslundii* *S. oralis*	Silver ions Vancomycin	Senthil and Çakir, 2024
**Alloplastic bone**						
Hydroxyapatites	Yes	Antibacterial effect (one of the most extensively studied)	*In vitro*	*P. gingivalis* *F. nucleatum* (efficent if mixed with BioOss) *Klebsiella pneumoniae* *Candida krusei* (0.2% w/w Ag) *E. coli* *B. subtilitis* (0.5% w/w Ag) *S. aureus* *Staphylococcus epidermidis* (1% w/w Ag) *Staphylococcus carnosus*	Silver, copper, zinc, strontium (do zrównoważenia cytotoksycznego działania jonów selenu), selenium	Kolmas et al., 2014; Gong et al., 2018; Maqbool et al., 2021; Li et al., 2023
Hydroxyapatite cements	Yes	Bacteriostatic Bactericidal	*In vitro*	*E. coli* *S. aureus*	Strontium ions Single study with Cu additive	Lv et al., 2022
Calcium phosphates	Yes	Bacteriostatic Bactericidal	*In vitro*	*E. faecium* *E. coli* *P. aeruginosa*	Silver, zinc ions	Fadeeva et al., 2021
Bioactive glass	Yes	Biocompatibility	*In vitro*	*E. coli* *S. aureus* *B. subtilis*	Zinc, copper, magnesium, strontium ions	Zamani et al., 2019; Anand et al., 2022
**Xenogeneic bone**						
Bio-Oss	Yes	Applied in defect filling after Actinomyces-associated lesion; antibacterial comparison with other materials	*In vivo, In vitro*	*Actinomyces* spp. *P. gingivalis* *F. nucleatum* (in mixture with AgHA)	Prophylactic clindamycin 300 mg ^*^TID × 7 days	Gong et al., 2018; Kaldas et al., 2020

^*^TID, three times a day.

Innovative materials combining demineralized bone matrix (DBM), calcium sulfate, and nanomaterials such as curcumin and silver nanoparticles (CU-NP and AgNP, respectively) have shown excellent antibacterial activity against *A. naeslundii* and *S. oralis* while maintaining favorable mechanical properties, highlighting their potential in maxillary bone regeneration (Senthil and Çakir, 2024). BioOss has demonstrated utility in peri-implantitis treatment and regenerative therapy for advanced periodontitis, though further long-term studies are needed (Esposito et al., 2012; Kaldas et al., 2020). Hydroxyapatites doped with silver, copper, strontium, or selenium, as well as bioactive glass with zinc, copper, or strontium, exhibit antimicrobial activity against Gram-positive and Gram-negative oral pathogens, maintain osteoblast biocompatibility, and support bone regeneration (Chen et al., 2007; Gong et al., 2018; Li Y. et al., 2022; Lv et al., 2022; Naruphontjirakul et al., 2023). Studies emphasize that the antimicrobial efficacy depends on ion concentration, with higher levels required for certain pathogens (e.g., 1% Ag for *S. aureus*), and that combinations of ions can balance cytotoxicity and enhance biological activity (Kolmas et al., 2014; Maqbool et al., 2021; Zheng et al., 2021; Anand et al., 2022; Li Y. et al., 2022; Weiss et al., 2023).

Cements and composites, such as silver-substituted tricalcium phosphate (Ag-TCP), zinc-substituted β-tricalcium phosphate, and multifunctional bioactive glasses, show promising antimicrobial activity against clinically relevant pathogens (*E. coli, S. aureus, Enterococcus faecium, P. aeruginosa*), with good biocompatibility and regenerative potential (Zamani et al., 2019; Honda et al., 2020; Fadeeva et al., 2021; Naruphontjirakul et al., 2023). These materials may reduce reliance on systemic antibiotics, contributing to safer and more effective bone regeneration and implant therapy. Nevertheless, *in vivo* studies are needed to evaluate bone quality, clinical outcomes, and the frequency of inflammatory complications.

In conclusion, contamination of grafts with saliva and oral bacteria is a key factor in postoperative infections, with *S. aureus* being the most common pathogen in bone infections. Alloplastic materials enhanced with metal ions (e.g., silver, copper) show significant antibacterial activity *in vitro* against oral bacteria and demonstrate superior resistance to infection compared to unmodified materials. Recent years have witnessed significant advances in the design of next-generation antibacterial bone substitute materials. These include nano-engineered composites, ion-doped ceramics, and bioactive polymer–ceramic hybrids that combine osteoconductive scaffolds with sustained antimicrobial release. Materials such as silver- and strontium-doped hydroxyapatite, copper- and zinc-enriched bioglasses, and chitosan-based calcium phosphate scaffolds have demonstrated dual activity—promoting osteogenesis while suppressing microbial biofilm formation (Fadeeva et al., 2021; Li et al., 2023; Naruphontjirakul et al., 2023). Table 6 summarizes representative studies highlighting emerging biomaterials with documented antibacterial and osteogenic properties. While antibiotic prophylaxis can reduce inflammatory complications and support bone regeneration, evidence does not consistently show additional benefit from extended antibiotic regimens, with “one-shot” prophylaxis often sufficient (Lyons et al., 2008; Milic et al., 2021; Remschmidt et al., 2023). Further research is necessary to optimize biomaterial composition, antimicrobial strategies, and clinical protocols for safe and effective bone regeneration in oral surgery.

**Table 6:** Summary of innovative antibacterial bone substitute materials.

**Material/composition**	**Antimicrobial mechanism**	**Osteogenic response**	**References**
Ag–Sr co-doped Hydroxyapatite	Ag^+^ disrupts membranes; Sr^2+^ enhances osteoblast activity	↑ ALP, ↑ mineralization	Li Y. et al., 2022
Cu–Zn bioactive glass	Ion release and ROS production	Promotes angiogenesis and bone formation	Kermani et al., 2023; Sharifianjazi et al., 2024
Chitosan/β-TCP composite	Cationic polymer–bacteria interaction	Enhances osteoblast adhesion	Ezati et al., 2018; Piaia et al., 2021
TiO_2_-Graphene oxide hybrid coating	Photocatalytic antibacterial effect	Osteoblast proliferation	Wang et al., 2020
Curcumin–Ag nanoparticle DBM composite	Synergistic antibacterial release	Maintains mechanical strength, biocompatible	Hassan et al., 2023; Hong et al., 2025
Sr/Fe-substituted hydroxyapatite in collagen hydrogel (Sr/Fe:HAp-Col)	Ciprofloxacin release with Sr^2+^/Fe^3+^ ions inhibits bacterial adhesion and inflammation	↑ ALP, ↑ Ca deposition, ↑ OPN, OCN, RUNX2 expression; enhanced *in vivo* bone formation in rat femur defect	Garbo et al., 2020
Shape-adaptive hydrogel with nano-HAp + Naringin for alveolar bone defects	Burst and sustained naringin release provides antibacterial effect; hydrogel degradation supports infection prevention	Enhances osteoblast differentiation and mineralization; effective in irregular and osteoporotic defects	Ullah et al., 2023; Tang et al., 2025
Copper-based biomaterials (e.g., Cu-doped ceramics)	Cu^2+^ ion release produces ROS and disrupts bacterial membranes	Promotes osteogenesis and angiogenesis at optimal ion concentrations	Kargozar et al., 2021; Shen et al., 2022; Li et al., 2025

## 5 Structure–property–function relationships in antibacterial biomaterials

The antibacterial efficacy of bone substitute materials is closely linked to their physicochemical attributes. Surface roughness, porosity, charge distribution, and ion release profiles govern bacterial adhesion and biofilm formation. For instance, increased nanoscale roughness enhances osteoblast attachment but simultaneously provides niches for microbial retention. Therefore, achieving a balance between osteoconductivity and antibacterial protection requires fine-tuning of surface topography and surface energy. Moreover, ion-doped materials (Ag^+^, Cu^2+^, Zn^2+^) prevent the adhesion of bacteria and the development of biofilms by release antimicrobial factors, that disrupt bacterial cell walls and inhibit quorum sensing while stimulating osteogenic differentiation through signaling pathways such as Wnt/β-catenin and BMP2. Composite materials incorporating polymers like chitosan or gelatin provide additional bacteriostatic barriers and control ion release kinetics. Understanding these structure–property–function interrelations will guide the rational design of future biomaterials with integrated antibacterial and regenerative functionalities (Yoda et al., 2014; Fosca et al., 2023; He et al., 2023; Wang et al., 2023; Kang et al., 2025; Kubiak-Mihkelsoo et al., 2025).

## 6 Conclusions and recommendation

Bone substitute materials play a pivotal role in oral and maxillofacial surgery, providing predictable outcomes in the reconstruction of alveolar bone defects and facilitating implant-supported rehabilitation. Microbial colonization and subsequent biofilm formation on graft surfaces constitute major determinants of clinical outcome, as they may delay healing, compromise integration, and predispose to graft failure. The susceptibility of a material to bacterial adhesion is largely determined by its physicochemical characteristics, including porosity, surface roughness, and wettability. Recent developments in biomaterial science, particularly the incorporation of antimicrobial agents such as silver, copper, zinc, and bioactive nanoparticles, offer promising strategies to mitigate microbial colonization while preserving osteogenic potential. Nonetheless, the majority of supporting evidence remains limited to *in vitro* studies, underscoring the need for robust *in vivo* and clinical investigations.

Based on available data it is recommended:

- Autogenous bone should be utilized whenever feasible; when alternative substitutes are indicated, the choice of material must consider not only osteogenic potential but also susceptibility to microbial colonization.
- Rigorous perioperative infection control protocols—including preoperative antiseptic rinses, appropriate single-dose antibiotic prophylaxis, and meticulous surgical closure—should be considered standard practice to minimize postoperative complications.
- Surgical techniques should aim to avoid graft exposure and ensure tension-free wound closure to limit microbial infiltration.
- Further development of biomaterials incorporating antimicrobial ions, nanoparticles, and bioactive coatings is strongly encouraged, with emphasis on optimizing antibacterial efficacy while ensuring cytocompatibility and long-term stability.
- Surface modifications that balance porosity for osteoconduction with reduced microbial adhesion should be prioritized in the design of future grafting materials.
- Longitudinal, randomized clinical trials are required to validate the antibacterial efficacy and regenerative potential of modified bone substitutes under clinical conditions.
- Standardized methodologies for assessing antibacterial properties of grafting materials should be established to allow reliable interstudy comparisons.
- Future investigations should focus on the host–material–microbiome interface to elucidate the immunological and microbiological mechanisms underlying graft integration and long-term clinical success.
